# Maintenance and Growth Requirements in Male Dorper × Santa Ines Lambs

**DOI:** 10.3389/fvets.2021.676956

**Published:** 2021-06-09

**Authors:** Marcilio S. Mendes, Jocely G. Souza, Caio Julio L. Herbster, Antonio S. Brito Neto, Luciano P. Silva, João Paulo P. Rodrigues, Marcos I. Marcondes, Ronaldo L. Oliveira, Leilson R. Bezerra, Elzania S. Pereira

**Affiliations:** ^1^Department of Animal Science, Federal University of Ceara, Fortaleza, Brazil; ^2^Department of Animal Science, Federal University of Southern and Southeastern Para, Xinguara, Brazil; ^3^Department of Animal Science, Washington State University, Pullman, WA, United States; ^4^Department of Animal Science, Federal University of Bahia, Salvador, Brazil; ^5^Department of Animal Science, Federal University of Campina Grande, Patos, Brazil

**Keywords:** crossbreed sheep, efficiency, energy, protein, warm areas

## Abstract

The aim of this study was to estimate the energy and protein requirements for maintenance and growth of lambs. A total of 35 crossbreed Dorper × Santa Ines lambs [31 ± 1.28 kg of initial body weight (BW) and 4 months old] were distributed in a completely randomized design with three treatments groups (*ad libitum*, 30 and 60% of feed restriction). Five lambs were slaughtered at the beginning of the experimental trial as a reference group to estimate the initial empty BW (EBW) and body composition. When the animals of the *ad libitum* treatment reached a BW average of 47.2 kg, at day 84 of trial, all lambs were slaughtered. The feed restriction promoted reduction in body fat (*P* < 0.001) and energy concentration (*P* < 0.001), while protein showed a quadratic response (*P* = 0.05). The equations obtained for NEg and NPg requirements were 0.2984 × EBW^0.75^ × EBWG^0.8069^ and 248.617 × EBW^−0.15546^, respectively. The net energy (NEm) and protein (NPm) for maintenance were 71.00 kcal/kg EBW^0.75^/day and 1.76 g/kg EBW^0.75^/day, respectively. In conclusion, the NEg and NPg requirement for lambs with 30 kg of BW and 200 g of average daily gain (ADG) were 0.736 Mcal/day and 24.38 g/day, respectively. Our findings indicate that the NEm for crossbreed Dorper × Santa Ines lambs is similar to those recommended by the international committees; however, we support the hypothesis that the requirements for gain are lower.

## Introduction

International committees (1–3) play an important role in establishing nutritional recommendations for sheep (4), especially for those in temperate regions. In tropical scenarios, the nutritional requirements recommended by international committees may not be adequate to meet the physiological needs at different stages of the animal's life (5). Evolutionary adaptations to the ecological opportunity of selective feeding in smaller animals, rather than by a physiological or metabolic necessity linked to body mass (6), may explain this phenomenon. Considerable efforts have been made to cluster scientific data and develop feeding systems for ruminants in warm regions (7). In Brazil, studies on the feed composition and nutritional requirements of hair sheep (5, 8, 9) have generated information to establish a committee to meet the real requirements of these animals (10). Warm regions are characterized by constantly high temperatures, sometimes associated with high humidity (3), which induces specificities in the characteristics of both feed and animals (8). Local breeds or crossbreed animals are often used in meat production systems in tropical regions (11, 12) and may present specific nutritional requirements (9, 13–15). Furthermore, an adequate supply of nutrients is necessary mainly to reduce protein costs and to reduce environmental pollution.

The objective of the present study was to determine the body composition and to estimate the energy and protein requirements for maintenance and growth in intact male Dorper × Santa Ines lambs using a comparative slaughter trial.

## Materials and Methods

### Site and Ethics Statement

The trial was conducted at the Animal Nutrition Laboratory of the Department of Animal Science of the Federal University of Ceara in Fortaleza, Ceara State, Brazil (30°43′02″S, 33°32′35″W). Throughout the trial period, the mean daily minimum and maximum air temperatures were 24.6°C ± 0.82 and 31.2°C ± 1.32, respectively, and the minimum and maximum relative humidity were 71.1% ± 7.58 and 89.1% ± 4.27, respectively. All procedures involving animal care and use were followed according to the standards established by the Ethics Committee on Animal Research of the Federal University of Ceara, Fortaleza, Brazil (UFC) (Protocol No. 3381260719).

### Experimental Design, Animal Management, and Diets

Thirty-five Dorper × Santa Ines intact male lambs, with average body weight (BW) of 31 ± 1.28 kg and 4 months old were distributed in a completely randomized design with three treatments groups (*ad libitum*, 30 and 60% of feed restriction). The diets were formulated with 14% crude protein (CP) to meet the requirements for a gain of 200 g/day (1). The total mixed ration (TMR) was composed of Tifton 85 hay, ground corn, soybean meal, dicalcium phosphate, and mineral premix (Table 1). The roughage:concentrate ratio was 60:40. The animals were identified, dewormed, and housed in individual pens (1.5 × 1.5 m) equipped with feed and water troughs. At the beginning of the trial, five lambs were randomly selected and slaughtered to serve as a reference group and used to estimate the initial empty BW (EBW) and body composition. The remaining animals were individually fed (diets as TMR) twice a day (at 0800 and 1600 h). The proportional supply of feed for animals in 30 and 60% feed restriction levels was calculated daily in relation to the average intake of animals subjected to *ad libitum* intake. Water was provided *ad libitum* for all animals. The lambs were weighed weekly to calculate the average daily gain (ADG). The trial lasted for 84 days.

**Table 1:** Ingredient proportions and chemical composition of total mix ration (TMR).

**Ingredient**	**g/kg dry matter (DM)**			
Tifton 85 grass hay	600.0			
Ground corn	260.7			
Soybean meal	124.0			
Dicalcium phosphate	10.2			
Mineral premix^a^	5.1			
**Nutrient** **(g/kg DM)**	**TMR**	**Tifton 85** **grass hay**	**Ground** **corn**	**Soybean** **meal**
Dry matter	911.0	916.4	906.6	900.2
Crude protein	141.5	86.1	70.2	508.4
Ether extract	30.0	26.7	44.3	13.6
Ash	65.5	70.2	16.3	64.4
Neutral detergent fiber	498.2	737.3	145.7	128.5
NDFap^b^	455.2	676.0	125.9	109.4
Acid detergent fiber	211.9	327.0	28.2	80.6
Total carbohydrate	763.0	817.0	869.2	413.6
Non-fibrous carbohydrate	307.8	141.0	743.4	304.2
Total digestible nutrients	624.5	–	–	–

a *Mineral premix was provided per kilogram of total diet DM, and the composition was as follow: 300–200 g of Ca, 50 g of P, 18 g of S, 40 g of Na, 16.5 g of Mg, 60 mg of Co, 85 mg of I, 2,000 mg of Mn, 11 mg of Se, 2,100 mg of Zn, 3,960 mg of Fe, 122 mg of Cu, 1,000 mg of Fl, 33.6 mg of vitamin A, 0.55 mg of vitamin D, 557.1 mg of vitamin E*.

b *NDFap, neutral detergent fiber corrected for ash and protein*.

### Calculations of Metabolizable Energy and Protein Intake

To evaluate the apparent total-tract digestibility of the dietary constituents and consequently the metabolizable energy intake (MEI), we performed a digestibility trial every 15 days during the experimental period by collecting feces for three consecutive days at specific times (9). The total digestible nutrient (TDN) was calculated according to Weiss (16). The MEI was estimated from TDN, where digestible energy (DE) was estimated as 4.409 Mcal/kg of TDN and converted to metabolizable energy (ME) using an efficiency of 82%, i.e., ME = 0.82 × DE (17).

Spot urine samples were collected every 15 days, approximately 4 h after the morning feeding, during spontaneous urination, used for analysis of purine derivatives to estimate microbial crude protein (MCP). The urine was homogenized, and a 5-ml sample was diluted in 45 ml 0.036 N sulfuric acid (1:10 ratio). The absorbed microbial purines and intestinal flow of microbial nitrogen were estimated from the equations proposed by Chen and Gomes (18). The MCP was calculated by multiplying the TDN intake (TDNI, kg/day) by the average of microbial efficiency of 135.5 g MCP/kg TDNI. The rumen degradable protein (RDP) was considered equal to MCP. The truly digestible microbial crude protein (tdMCP) was estimated by the followed equation:

tdMCP=(135.5×TDNI)×0.64

where TDNI = TDN intake, and 0.64 is the value considering that the MCP is constituted of 80% of amino acids with intestinal digestibility of 80% (17).

The rumen undegradable protein (RUP) intake was calculated as CP intake minus RDP. The digestible rumen undegradable protein (dRUP) was calculated according to the followed equation:

dRUP=RUP×0.80

where 0.80 is the fixed value of 80% in digestibility of RUP in the small intestine (17). The metabolizable protein intake (MPI) was calculated as the sum of the tdMCP and dRUP.

### Slaughter, Sampling, and Chemical Analyses

When the *ad libitum* group reached a BW average of 47.2 kg, all animals were slaughtered. Before slaughter, fasted BW (FBW) was determined as the BW after 18 h of no access to feed and water. At slaughter, lambs were stunned with a captive pistol, followed by severing of the jugular vein and carotid artery. Blood collection procedures, gastrointestinal tract, organs, and other parts of the body were performed followed as described by Pereira et al. (8). The EBW was calculated by subtracting the weight of gastrointestinal and bladder contents from FBW. The carcasses were refrigerated at 4°C for 24 h and then were divided into right and left half-carcasses. Subsequently, the right half-carcasses, non-carcass components (blood, head, hooves, internal organs, and the cleaned gastrointestinal tract), and hides were frozen and then cut with a band saw and ground in an industrial cutter. After grinding and homogenization, samples of 500 g were taken and then frozen at −20°C. The samples were placed in a forced-ventilation oven at 55°C for 72 h, after which they were defatted by extraction with ether in a Soxhlet apparatus for 12 h, method number 920.39 (19). Afterwards, they were ground in a ball mill for the subsequent chemical analyses of body composition. The dry matter (DM), ash, and CP content levels were determined by fat-free samples, following the methods described below for experimental ingredients diets. The body water content was calculated as 100% minus DM.

For analysis, feed, orts, and fecal samples were dried in a forced-air oven at 55°C for 72 h and then ground in a Wiley mill (TE-650; Tecnal, Piracicaba, São Paulo, Brazil) with a 1-mm sieve. The DM (method 967.03), CP (method 981.10), ash (method 942.05), ether extract (EE, method 920.39), and acid detergent fiber (ADF, method 913.18) were conducted as described by the Association of Official Analytical Chemists (19). The neutral detergent fiber (NDF) content was performed as described by Van Soest et al. (20) using thermostable alpha-amylase without sodium sulfite and corrected for residual ash (21) and residual nitrogenous compounds (22). The total carbohydrate content was calculated according to (23), and nonfibrous carbohydrates were calculated using an equation adapted from Weiss (16).

### Models and Calculations

To estimate EBW (kg) and EBW gain (EBWG, kg/day), equations obtained from the linear regression of the FBW against the BW, EBW against the FBW and EBWG against the ADG were generated. Only performance animals were used to develop the EBWG equation.

The empty body weight energy (BEC) content was obtained from the body contents of protein (EBP) and fat (EBF) and their respective caloric equivalents of 5.6405 and 9.3929 Mcal/kg (24).

The retained energy (RE) was obtained as the difference between final and initial body energy contents. The initial body energy contents were estimated from the reference group data by regressing body energy content on EBW.

The net energy requirement for weight gain (NEg, Mcal/day) was estimated using the model used by Chizzotti et al. (25):

$$\text{NEg}=\beta_{0}\times {\text{EBW}}^{0.75}\times {\text{EBWG}}^{\beta_{1}\;}$$

where β_0_ and β_1_ = coefficients obtained from the regression of the logarithm of RE (Mcal/kg EBW^0.75^/day) against the logarithm of EBWG (kg/day).

Heat production (HP) was calculated as the difference between MEI and RE. The net energy requirement for maintenance (NEm) was assumed to be the intercept (β_0_) of the exponential regression between HP and MEI as proposed by Ferrell and Jenkins (26):

$$\text{HP}=\beta_{0}\times {\text{e}}^{\left(\beta_{1}\times \text{MEI}\right)}$$

where HP and MEI are expressed in Mcal/kg EBW^0.75^/day, and β_1_ is the equation parameter.

The metabolizable energy requirements for maintenance (MEm), expressed as Mcal/kg EBW^0.75^/day, were estimated by the iterative method as the point where MEI is equal to HP (i.e., the point at which there is no energy retention in the body). In addition, the efficiency of use of metabolizable energy for maintenance (k_m_) was estimated by the ratio between NEm and MEm.

The efficiency of metabolizable energy use for gain (k_g_) was considered the slope (β_1_) of the regression of the RE against MEI:

$$\text{RE}=\beta_{0}+\beta_{1}\times \text{MEI}$$

where RE = retained energy (Mcal/kg EBW^0.75^/day), MEI = metabolizable energy intake (Mcal/kg EBW^0.75^/day), and β_0_ = the equation parameter.

To calculate the net protein requirements for any body weight gain (NPg, g/day), we adjusted the following model:

$$\text{NPg}=\beta_{1}\times 10^{\beta_{0}}\times {\text{EBW}}^{\left(\beta_{1}-1\right)}$$

where β_0_ and β_1_ = regression parameters. Reference and performance animals were included in this model.

To estimate the net protein requirements for maintenance (NPm, g/kg EBW^0.75^/day), the retained protein was plotted as a function of MPI according following equation:

$$\text{RP}=\beta_{0}+\beta_{1}\times \text{MPI}$$

where RP = retained protein (g/kg EBW^0.75^/day), MPI = metabolizable protein intake (g/kg EBW^0.75^/day), β_0_ = NPm, and β_1_ = k_pg_.

The metabolizable protein requirement for maintenance (MPm) was obtained from the adaptation of the Wilkerson et al. (27) and National Research Council (NRC) (17) equations. MPI was related to the EBWG of the lambs according to the following equation:

$$\text{MPI}=\beta_{0}+\beta_{1}\times \text{EBWG}$$

where MPI = MP intake (g/day), EBWG = EBW gain (kg/day), and β_0_ and β_1_ = parameters determined from a linear regression. Posteriorly, the ratio between the intercept (β_0_) and the average EBW^0.75^ of the lambs was considered as MPm:

$$\text{MPm}=\frac{\beta_{0}}{{\text{EBW}}^{0.75}}$$

The efficiency of metabolizable protein use for maintenance (k_pm_) was calculated as NPm/MPm.

The estimated requirements based on the EBW were converted to the FBW using the factor (1.14), which was obtained from the ratio FBW^0.75^/EBW^0.75^.

### Statistical Analysis

A linear model analysis was performed following a completely randomized design. The statistical model is Yij = μ + αi + eij, where Yij = value observed that received treatment i, μ = overall mean, αi = fixed effect of treatment i; eij = random error ~ NID (0, σ2).

Treatments were analyzed as orthogonal partition into linear and quadratic effects. A significance level of 5% (α = 0.05) was adopted in this study. We carried out all analysis at SAS System Software (SAS 9.0, SAS Institute Inc., Cary, NC, USA; 2003), ANOVA with generalized linear model (GLM) procedure, linear regressions with REG procedure, and nonlinear models using NLIN procedure along with Marquardt iterative method.

## Results

### Performance, Intake, Energy Retention, and Body Composition

The final BW, ADG, EBW, and EBWG showed a linear response by feed restriction (*P* < 0.001). The generated equations to predict EBW and EBWG for all experimental animals were FBW (kg) = 0.756 (±0.659) + 0.912 (±0.017) × BW [R^2^ = 0.99; root mean square error (RMSE) = 0.673]; EBW (kg) = 0.547 (±0.564) + 0.827 (±0.016) × FBW (R^2^ = 0.99; RMSE = 0.631); EBWG (kg/day) = 0.043 (±0.011) + 0.590 (±0.054) × ADG (R^2^ = 0.94; RMSE = 0.006).

The DMI (kg/day; g/kg EBW^0.75^/day), MEI, RE, HP (Mcal/kg EBW^0.75^/day), fat (%EBW), and energy (%EBW) decreased linearly with increased feed restriction (*P* < 0.001); however, the protein (%EBW) showed a quadratic response by feed restriction (*P* = 0.05) (Table 2).

**Table 2:** Effects of feed restriction on performance, intake and energy retention, and body composition in intact males Dorper × Santa Ines lambs.

**Parameters**	**REF**	**SEM**	**Treatments groups**			**SEM**	***P*****-value**	
			**AL**	**30%**	**60%**		**Linear**	**Quadratic**
Initial BW (kg)	31.2	2.70	31.0	31.8	31.9	0.400	0.124	0.532
Final BW (kg)	–	–	47.2	40.5	29.3	0.733	<0.001	0.020
FBW (kg)	29.2	1.95	40.9	36.0	27.0	0.604	<0.001	0.010
ADG (g/day)	–	–	192.2	103.7	−31.0	10.578	<0.001	0.086
EBW (kg)	24.3	1.33	30.6	28.1	24.0	0.288	<0.001	0.040
EBWG (g/day)	–	–	152.5	84.3	−13.6	7.091	<0.001	0.100
**Intake and energy balance**								
DMI (kg/day)	–	–	1.369	0.972	0.513	0.033	<0.001	0.454
DMI (g/kg EBW^0.75^/day)	–	–	104.9	79.6	47.2	1.998	<0.001	0.160
CPI (g/kg EBW^0.75^/day)	–	–	15.3	11.1	6.6	0.283	<0.001	0.734
MEI (Mcal/kg EBW^0.75^/day)	–	–	0.231	0.180	0.110	0.005	<0.001	0.144
RE (Mcal/kg EBW^0.75^/day)	–	–	0.04	0.02	0.00	0.003	<0.001	0.262
HP (Mcal/kg EBW^0.75^/day)	–	–	0.189	0.155	0.111	0.005	<0.001	0.363
**Body composition**								
Water (%EBW)	65.65	2.31	60.40	61.06	62.36	0.735	0.071	0.720
Protein (%EBW)	18.46	0.74	16.53	17.99	17.42	0.405	0.131	0.050
Fat (%EBW)	12.26	2.63	19.09	16.46	14.62	0.840	<0.001	0.705
Ash (%EBW)	3.57	0.36	3.63	3.99	4.21	0.126	0.003	0.670
Energy (Mcal/kg EBW)	2.19	0.25	2.72	2.56	2.36	0.069	<0.001	0.811

*REF, reference group; AL, ad libitum intake; 30% and 60%, feed restriction; BW, body weight; FBW, fasted body weight; ADG, average daily gain; EBW, empty body weight; EBWG, empty body weight gain; DMI, dry matter intake; CPI, crude protein intake; MEI, metabolizable energy intake; RE, retained energy; HP, heat production; SEM, standard error of the mean*.

*P-value for treatment effect without the reference group*.

The BFC and BEC increased with increasing BW; however, BPC decreased with increasing BW (Table 3).

**Table 3:** Body fat, protein, and energy contents of intact male Dorper × Santa Ines lambs from 30 to 50 kg BW.

**BW**	**EBW**	**BFC**	**BPC**	**BEC**
**(kg)**	**(kg)**	**(g/kg EBW)**	**(g/kg EBW)**	**(Mcal/kg EBW)**
30	28.17	134.23	179.81	2.30
35	27.61	150.20	175.75	2.43
40	31.39	165.65	172.28	2.55
45	35.17	180.70	169.26	2.66
50	38.94	195.32	166.60	2.77

*BW, body weight; EBW, empty body weight; BFC, body fat content; BPC, body protein content; BEC, body energy content*.

### Energy and Protein Requirements

**Figure 1:**
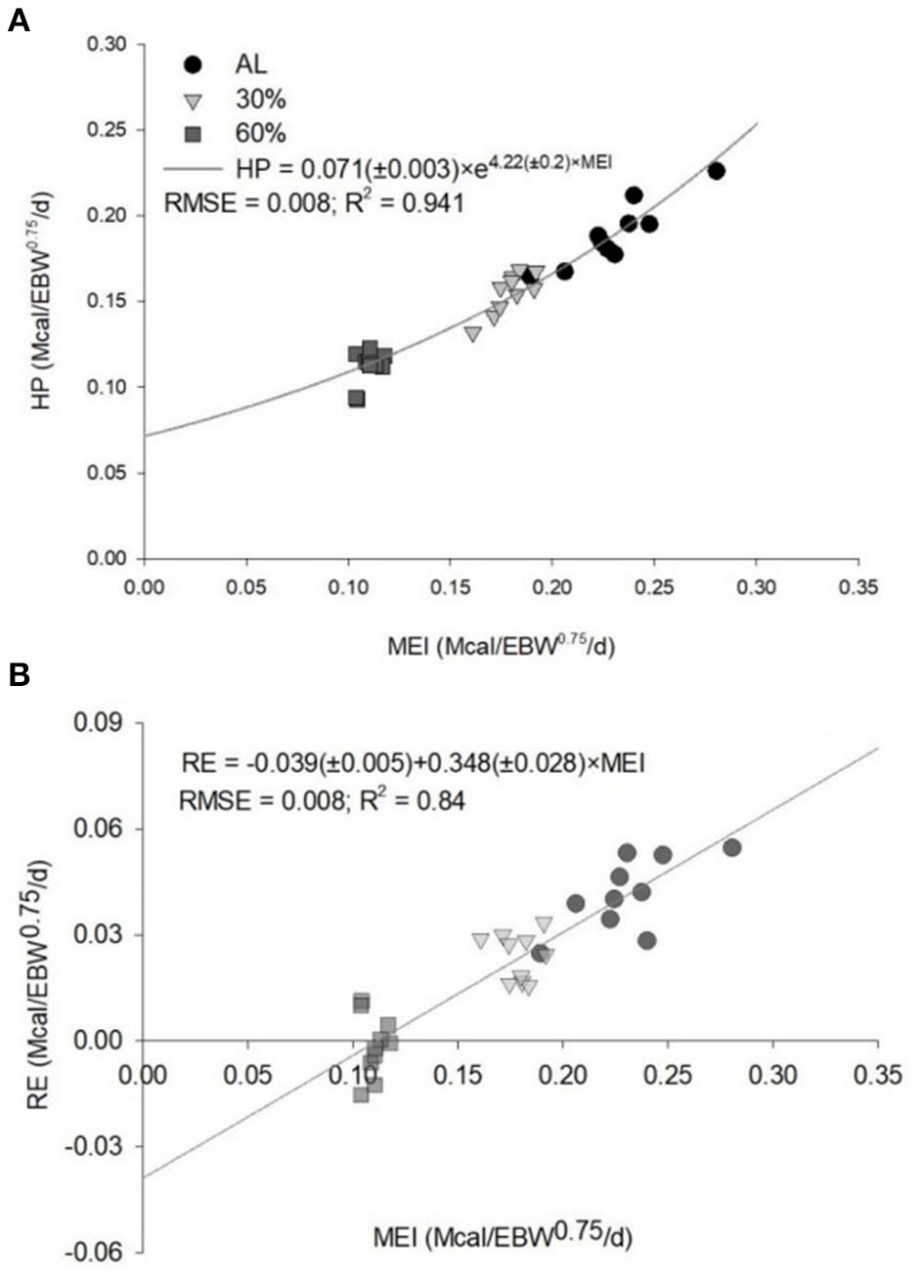
Predicted equations by the relationships **(A)** between the heat production (HP) and the metabolizable energy intake (MEI) and **(B)** between the retained energy (RE) and the MEI of *ad libitum* (AL), 30% of feed restriction (30%) and 60% of feed restriction (60%) of intact male Dorper × Santa Ines lambs. EBW, empty body weight; RMSE, root mean square error.

The equation generated to estimate the NEg (Mcal/kg EBW^0.75^/day) was 0.2984 × EBW^0.75^ × EBWG^0.8069^. The NEg estimated for intact male Dorper × Santa Ines lambs were 0.736 Mcal/day, considering a BW of 30 kg and ADG of 200 g/day (Table 4). The k_g_ obtained was 0.348. The value of HP when MEI is zero (NEm) was estimated to be 0.071 Mcal/kg EBW^0.75^/day (Figure 1). The MEm was 0.115 Mcal/kg EBW^0.75^/day, and the k_m_ was 0.61.

**Table 4:** Net energy and protein requirements for weight gain in intact male Dorper × Santa Ines lambs from 30 to 50 kg BW.

**BW (kg)**	**EBW (kg)**	**ADG (g/day)**			
		**100**	**150**	**200**	**250**
**Energy (Mcal/day)**					
30	28.17	0.508	0.624	0.736	0.843
35	27.61	0.567	0.697	0.821	0.941
40	31.39	0.625	0.768	0.904	1.036
45	35.17	0.680	0.836	0.985	1.128
50	38.94	0.734	0.902	1.063	1.218
**Protein (g/day)**					
30	28.17	15.14	19.89	24.38	28.86
35	27.61	15.06	19.44	23.82	28.21
40	31.39	14.77	19.06	23.35	27.65
45	35.17	14.51	18.73	22.95	27.16
50	38.94	14.28	18.43	22.58	26.74

*BW, body weight; EBW, empty body weight; ADG, average daily gain*.

**Figure 2:**
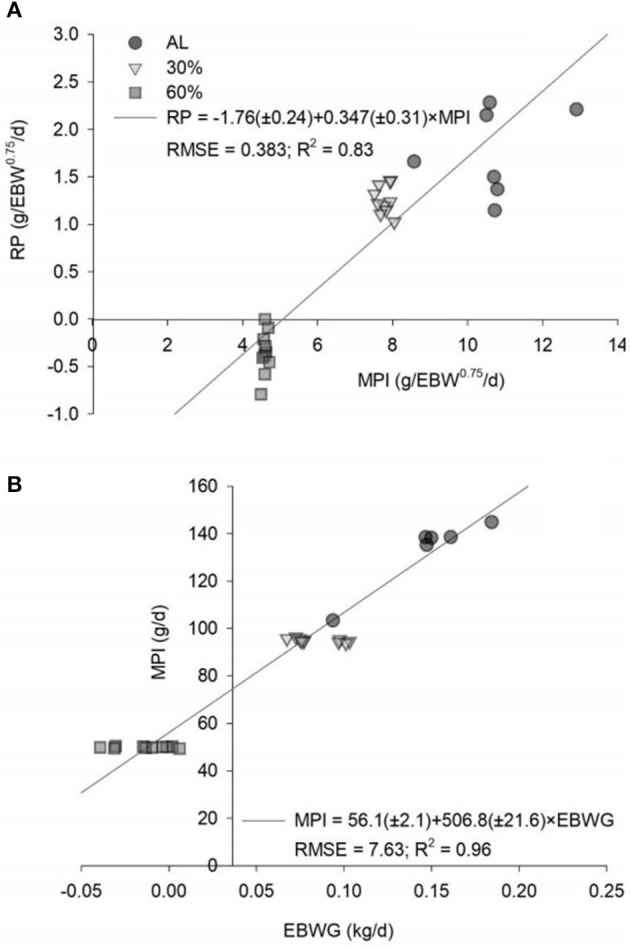
Predicted equations by the relationships **(A)** between the retained protein (RP) and the metabolizable protein intake (MPI) and **(B)** between the MPI and the empty BW gain (EBWG) of *ad libitum* (AL), 30% of feed restriction (30%) and 60% of feed restriction (60%) of intact male Dorper × Santa Ines lambs. EBW, empty body weight; RMSE, root mean square error.

The equation to estimate the NPg (g/day) was NPg = 248.617 × EBW^−0.15546^. The NPg was 24.38 g/day, considering a BW of 30 kg and an ADG of 200 g/day (Table 4). The relationship between the MPI and the EBWG is show in Figure 2. The MPm was 4.31 g/kg EBW^0.75^/day. The NPm was 1.76 g/kg EBW^0.75^/day, and the k_pg_ was 0.347 (Figure 2).

## Discussion

Brazilian studies with hair sheep have generated a considerable amount of data that has contributed to improving our understanding of nutritional requirements. Nutrient requirements are not static (4) and vary with genetic selection (11) and crossbreeding (28). Factors such as mathematical models (29), environmental conditions (5), genotype (30) body composition, and feed quality (9) may influence the NEm requirements.

The body composition and body part masses were predicted for a wide range of live weights, that is, from 30 to 50 kg of Dorper × Santa Ines. In the absence of specific estimates in the literature, the results of this study might be useful for predicting the protein and energy requirements for this category. The NEm obtained in our study was 0.071 Mcal/kg EBW^0.75^/day (or 0.062 Mcal/kg FBW^0.75^/day). This value is 16.9% greater (0.059 Mcal/kg EBW^0.75^/day) in relation to the value obtained with hair sheep by Oliveira et al. (5). The value determined in this study was consistent with the unadjusted energy requirement value of the NRC (1), which is 0.062 Mcal/kg FBW^0.75^/day. The crossbreed Dorper × Santa Ines lamb has early maturing when compared to Santa Ines (8, 13) and Morada Nova (9) breeds. The Dorper genotype presents a fast development (11), and it may influence the NEm result. We also verified that MEm was consistent with those reported by NRC (1) (0.102 vs. 0.096 Mcal/day, respectively). It is known that, for the same gain, protein and energy cost may be different depending on the body composition (31). Energetically, fat deposition is more efficient than protein deposition due to the different biochemical pathways and the greater daily turnover of protein than fat (32). The efficiency use of ME for protein deposition (k_p_) ranges from 10 to 40%, as the efficiency use of ME for fat deposition (k_f_) ranges from 60 to 80% (33).

It is reported that ME requirements of ruminants raised in tropical regions are higher than the published values for temperate genotypes (15). However, the expression of the allometric relationship that smaller species requiring more per unit body weight, while mathematically correct, would only explain anything if it was shown that some other factor relates directly to unit body weight. The statement that smaller animals have higher mass-specific metabolic requirements than large animals express the same fact as the statement that smaller animals have the same metabolic requirements as large animals on a metabolic body weight basis (note that the allometric relationship also allows to correctly state that smaller animals have lower absolute metabolic requirements than large animals).

The NEg requirements obtained in our study were lower than those recommended by the NRC (1) for 4-month-old early maturing lambs. Thus, for a lamb with 30 kg of BW and ADG of 200 g/day, the NEg requirement estimated in the current study was 0.736 Mcal/day, 19% lower than that estimated by NRC (1) (0.910 Mcal/day). As well as NEg estimates, k_g_ values may be affected by the composition of weight gain, so that in sheep, values between 0.18 and 0.30 and 0.66 and 0.74 have been reported for protein deposition and fat, respectively (14, 34). In our study, the k_g_ was 0.348. The Small Ruminant Nutrition System (SRNS) uses k_g_ estimated from the proportion of energy retained in the form of protein, considering 0.27 and 0.68 for protein and fat, respectively (35). Higher values of k_g_ of 0.345 and 0.409 for animals fed with medium- and low-quality forage, respectively, were reported for crossbreed animals Dorper × Santa Ines. These differences being greater associated with changes in the efficiency of fat deposition (14).

In comparative terms, the MPm of 3.78 g/kg BW^0.75^/day is higher than NRC (1) of 3.27 g/kg BW^0.75^/day. Our estimates are similar to those reported by Wilkerson et al. (27) for beef cattle (3.8 g/kg BW^0.75^/day). For all types of growing goats, MPm of 3.07 g/kg BW^0.75^/day was reported by Luo et al. (36). Higher requirements for MPm can be attributed to the high rates of metabolism of visceral organs and tissues during the growth of the animal, which increases maintenance costs compared to animals that have reached maturity weight (37). Differences in the MP requirements are attributed to dietary quality. The contribution of MCP to the MP intake in our study was computed as 135.5 g MCP/kg TDN intake. Therefore, the estimate of 3.78 g/kg BW^0.75^/day is not independent of the estimate of MCP, meaning that 3.78 g/kg BW^0.75^/day is valid only when the 135.5 g MCP/kg TDN intake is used to predict MCP. Animals fed roughages of low nutritional value tend to have low N retention and consequently higher protein requirements. In our study, the k_pm_ obtained was 0.41, which is lower than the values adopted by the international committees. However, the great variability that exists between the values adopted [0.75 for Agricultural Research Council (ARC) (24); 0.67 for Commonwealth Scientific and Industrial Research Organization (CSIRO) (37), and NRC (1); 1.0 for Agricultural and Food Research Council (AFRC) (38, 39); and 0.70 for CSIRO (2)] illustrates the uncertainty about the actual efficiency of use of the absorbed amino acids (AA). The efficiency of use of the metabolizable protein depends on the source of MP for the synchronization between the AA profile of the metabolized protein and the maintenance-related tissues; therefore, it is positively correlated with the protein biological value (40). In addition, the estimates of MPI can contribute to the variability observed in the efficiency of protein use. An inaccuracy associated with the mathematical models used to estimate the intake of RUP and the constant values used to calculate the digestible fractions of the true microbial protein and RUP (0.80) may contribute to the underestimation or overestimation of MPI. Differences in the need for metabolizable proteins can be attributed to the quality of the diet (15). Given the uncertainties in the determination of MCP, current estimates of metabolizable protein required for maintenance are biased. The use of empirical equations to predict MCP, which, in turn, is used to estimate metabolizable protein intake, is risky because it establishes a dependency between these estimates and creates a specificity that is not appropriate for mechanistic systems. Despite the existence of data and knowledge about the partitioning of retained energy into fat and protein, the prediction of retained protein remains unsatisfactory.

In our study, the NPg decreased as the lambs' body weight increased. The reduction in BW protein concentration with advancing maturity has been clearly established in sheep (41) and determines the decrease in daily requirements for weight gain. As the animal grows, total protein and ash content increase at similar rates in early life decelerating later. The NPg requirements are represented by the amount of AA made available to the animal tissue, discounting the AA pool that is metabolically prioritized by the animal to counteract the endogenous N losses by the animal's organism, such as losses of CP in feces, urine, wool, and/or scurf and fiber (1). The NPg estimates presented in the NRC (1) are higher than the estimates obtained for Dorper × Santa Ines sheep. The high rate of body fat deposition reported by the NRC (1) differs from our estimates. Many factors can alter the gain composition during the feeding period, but it is assumed that the composition of the fat-free matter remains constant. Similarly, the heats of combustion of fat and protein are assumed to be invariable, although lower differences might exist as a result of differences in the determination. The k_pg_ estimated in the current study (0.35) was higher than those obtained in hair sheep trials (8, 9). As with the efficient use of metabolizable protein for maintenance, there is no consensus regarding the values of k_pg_, which have varied between 0.59 (39) and 0.70 (1, 2, 37), the k_pg_ obtained with Dorper × Santa Ines sheep is compatible with the idea that the efficiency of use of the metabolizable protein is influenced by the energy supply to the animals, which, possibly, is associated with the reduction in the use of AA for hepatic gluconeogenesis where energy intake is high.

The use of recommendations based on international feeding systems has as consequence nutrient wastage for Dorper × Santa Ines between 30 and 50 kg of body weight, since the amounts of energy and protein required for the gain of the sheep were lower than the values recommended by international committees. These findings are of great importance for the targeted improvement of nutrient levels in ruminants. In conclusion, the net and metabolizable energy requirements for maintenance of crossbreed Dorper × Santa Ines lambs were 71.00 and 115.00 kcal/kg EBW^0.75^/day, respectively. The net energy and protein requirements for gain could be obtained by the respective equations NEg (Mcal/day) = 0.2984 × EBW^0.75^ × EBWG^0.8069^ and NPg (g/day) = 248.617 × EBW^−0.15546^.

## Data Availability Statement

The raw data supporting the conclusions of this article will be made available by the authors, without undue reservation.

## Ethics Statement

The animal study was reviewed and approved by Ethics Committee on Animal Research of the Federal University of Ceara, Fortaleza, Brazil (UFC) (Protocol number 3381260719).

## Author Contributions

ESP is the leader of the research project and responsible for all parts of the study, from the project to the publication. MSM, CJLH, and ASBN contributed to performing the experiment and collecting the data. ESP, JGS, JPPR, RLO, and LRB contributed to the writing, review, and editing of the manuscript. JPPR, MIM, and LPS analyzed the data. All authors contributed to the article and approved the submitted version.

## Conflict of Interest

The authors declare that the research was conducted in the absence of any commercial or financial relationships that could be construed as a potential conflict of interest.

## References

[1] NRC. Nutrient Requirements of Small Ruminants: Sheep, Goats, Cervids and New World Camelids. Washington, DC: National Academy Press (2007).

[2] CSIRO. Nutrient Requirements of Domesticated Ruminants. Collingwood: CSIRO Publishing (2007).

[3] INRA. INRA Feeding System for Ruminants. Wageningen: Wageningen Academic Publishers (2018).

[4] Cannas A Tedeschi LO Fox DG Pell AN Van Soest PJ. A mechanistic model for predicting the nutrient requirements and feed biological values for sheep. J Anim Sci. (2004) 82:149–69. 10.2527/2004.821149x 14753358

[5] Oliveira AP Pereira ES Biffani S Medeiros AN Silva AMA Oliveira RL. Meta-analysis of the energy and protein requirements of hair sheep raised in the tropical region of Brazil. J Anim Physiol Anim Nutr. (2017) 102:E52–60. 10.1111/jpn.12700 28252227

[6] Clauss M Steuer P Müller DW Codron D Hummel J. Herbivory and body size: allometries of diet quality and gastrointestinal physiology, and implications for herbivore ecology and dinosaur gigantism. PLoS ONE. (2013) 8:E68714. 10.1371/journal.pone.0068714 24204552 PMC3812987

[7] BR-CORTE. Nutrient Requirements of Zebu and Crossbred Cattle. 3rd.Edn. Viçosa: Suprema Gráfica Ltda (2016). 10.5935/978-85-8179-111-1.2016B002

[8] Pereira ES Lima FWR Marcondes MI Rodrigues JPP Campos ACN Silva LP. Energy and protein requirements of Santa Ines lambs, a breed of hair sheep. Animal. (2017) 11:2165–74. 10.1017/S1751731117001185 28578721

[9] Pereira ES Pereira MWF Marcondes MI Medeiros NA Oliveira RL Silva LP. Maintenance and growth requirements in male and female hair lambs. Small Rumin Res. (2018) 159:75–83. 10.1016/j.smallrumres.2017.11.00326431709

[10] Herbster CJL Silva LP Marcondes MI Garcia IFF Oliveira R Cabral L. Weight adjustment equation for hair sheep raised in warm conditions. Animal. (2020) 14:1718–23. 10.1017/S1751731120000294 32148215

[11] Malhado CHM Carneiro PLS Affonso PRAM Souza AAO Sarmento JLR. Growth curves in Dorper sheep crossed with the local Brazilian breeds, Morada Nova, Rabo Largo, and Santa Inês. Small Rumin Res. (2009) 84:16–21. 10.1016/j.smallrumres.2009.04.006

[12] Souza DA Villarroel ABS Pereira ES Osório JCS Teixeira A. Growth performance, feed efficiency and carcass characteristics of lambs produced from Dorper sheep crossed with Santa Inês or Brazilian Somali sheep. Small Rumin Res. (2013) 114:51–5. 10.1016/j.smallrumres.2013.06.006

[13] Regadas Filho JGL Pereira ES Pimentel PG Villarroel ABS Medeiros AN Fontenele RM. Body composition and net energy requirements for Santa Ines lambs. Small Rumin Res. (2013) 109:107–12. 10.1016/j.smallrumres.2012.07.01128578721

[14] Galvani DB Pires AV Susin I Gouvêa VN Berndt A Chagas LJ. Energy efficiency of growing ram lambs fed concentrate-based diets with different roughage sources. J Anim Sci. (2014) 92:250–63. 10.2527/jas.2012-6017 24352972

[15] Salah N Sauvant D Archimède H. Nutritional requirements of sheep, goats and cattle in warm climates: a meta-analysis. Animal. (2014) 8:1439–47. 10.1017/S1751731114001153 24902005

[16] Weiss WP. Symposium: prevailing concepts in energy utilization by ruminants. Predicting energy values of feeds. J Dairy Sci. (1993) 76:1802–11. 10.3168/jds.S0022-0302(93)77512-8

[17] NRC. Nutrient Requirements of Beef Cattle, 7 Edn. Washington, DC: National Academy Press (2000).

[18] Chen XB Gomes JM. Estimation of microbial protein supply to sheep and cattle based on urinary excretion of purine derivatives: an overview of the technical details. Occasional Publication of the International Feed Resources Unit. Rowett Research Institute. Bucksburn, Aberdeen (1992).

[19] AOAC. Official Methods of Analysis, 15th Edn. Washington, DC: AOAC International (1990).

[20] Van Soest PJ Robertson JB Lewis BA. Methods for dietary fiber, neutral detergent fiber, and non-starch polysaccharides in relation to animal nutrition. J Dairy Sci. (1991) 74:3583–97. 10.3168/jds.S0022-0302(91)78551-2 1660498

[21] Mertens DR Allen M Carmany J Clegg J Davidowicz A Drouches M. Gravimetric determination of amylase-treated neutral detergent fiber in feeds with refluxing in beakers or crucibles: collaborative study. J AOAC Int. (2002) 85:1217–40. 12477183

[22] Licitra G Hernandez TM Van Soest PJ. Standardization of procedures for nitrogen fractionation of ruminant feeds. Anim Feed Sci Tech. (1996) 57:347–58. 10.1016/0377-8401(95)00837-3

[23] Sniffen CJ O'Connor JD Van Soest PJ Fox DG Russell JB. A net carbohydrate and protein system for evaluating cattle diets: II. carbohydrate and protein availability. J Anim Sci. (1992) 70:3562–77. 10.2527/1992.70113562x 1459919

[24] ARC. The Nutrient Requirement of Ruminant Livestock. Slough: Commonwealth Agricultural Bureaux (1980).

[25] Chizzotti ML Tedeschi LO Valadares Filho SC. A meta-analysis of energy and protein requirements for maintenance and growth of Nellore cattle. J Anim Sci. (2008) 86:1588–97. 10.2527/jas.2007-0309 18375666

[26] Ferrell CL Jenkins TG. Body composition and energy utilization by steers of diverse genotypes fed a high-concentrate diet during the finishing period: II. Angus, Boran, Brahman, Hereford, and Tuli sires. J Anim Sci. (1998) 76:647–57. 10.2527/1998.762647x 9498376

[27] Wilkerson VA Klopfenstein TJ Britton RA Stock RA Miller PS. Metabolizable protein and amino acid requirements of growing cattle. J Anim Sci. (1993) 71:2777–84. 10.2527/1993.71102777x 8226380

[28] Nsahlai IV Goetsch AL Luo J Johnson ZB Moore JE Sahlu T. Metabolizable energy requirements of lactating goats. Small Rumin Res. (2004) 53:253–73. 10.1016/j.smallrumres.2004.04.007

[29] Tedeschi LO Fox DG Guiroy PJ. A decision support system to improve individual cattle management. 1. A mechanistic, dynamic model for animal growth. Agric Syst. (2004) 79:171–204. 10.1016/S0308-521X(03)00070-2

[30] Teixeira IAMA Fernandes MHMR Filho JMP Canesin RC Gomes RA Resende KT. Body composition, protein and energy efficiencies, and requirements for growth of F1 Boer x Saanen goat kids. J Anim Sci. (2017) 95:2121–32. 10.2527/jas.2016.1252 28726997

[31] Galvani DB Pires CC Kozloski GV Wommer TP. Energy requirements of Texel crossbred lambs. J Anim Sci. (2008) 86:3480–90. 10.2527/jas.2008-1097 18708598

[32] Vermorel M Bickel H. Utilization of feed energy by growing ruminants. Ann Zootech. (1980) 29:127–43.

[33] Garrett WN. Factors influencing energetic efficiency of beef production. J Anim Sci. (1980) 51:1434–40. 10.2527/jas1981.5161434x

[34] Graham RM. Variation in energy and nitrogen utilization by sheep between weaning and maturity. Austr J Agric Res. (1980) 31:335–45.

[35] Tedeschi LO Cannas A Fox DG. A nutrition mathematical model to account for dietary supply and requirements of energy and other nutrients for domesticated small ruminants: the development and evaluation of the Small Ruminant Nutrition System. Small Rumin Res. (2010) 89:174–84. 10.1016/j.smallrumres.2009.12.041

[36] Luo J Goestch AL Nsahlai IV Sahlu T Ferrell CL Owens FN. Metabolizable protein requirements for maintenance and gain of growing goats. Small Rumin Res. (2004) 53:309–26. 10.1016/j.smallrumres.2004.04.003

[37] CSIRO. Feeding Standards for Australian Livestock. Ruminants. Melbourne: CSIRO Publications (1990).

[38] AFRC. Nutritive Requirements of Ruminant Animals: Protein (Report 9). Nutr Abstr Rev. (1992) 62:787–835.

[39] AFRC. Energy and Protein Requirements of Ruminants. Wallingford: CAB International (1993).

[40] Cantalapiedra-Hijar G Ortigues-Marty I Sepchat B Agabriel J Huneau JF Fouillet H. Diet–animal fractionation of nitrogen stable isotopes reflects the efficiency of nitrogen assimilation in ruminants. Br J Nutr. (2015) 113:1158–69. 10.1017/S0007114514004449 25716533

[41] Galvani DB Pires CC Kozloski GV Sanchez LMB. Protein requirements of texel crossbred lambs. Small Rumin Res. (2009) 81:55–62. 10.1016/j.smallrumres.2008.11.003

